# Do all anatomic stems perform equally at long-term survival? A regional registry-based study on 12,010 total hip arthroplasty implants according to stem length and neck modularity

**DOI:** 10.1186/s10195-025-00824-3

**Published:** 2025-02-21

**Authors:** Alberto Di Martino, Valentino Rossomando, Barbara Bordini, Matteo Brunello, Riccardo Ferri, Cesare Faldini

**Affiliations:** 1 Department of Orthopedic and Traumatology, IRCCS Istituto Ortopedico Rizzoli, Via Giulio Cesare Pupilli 1, Bologna, Italy; 2 Department of Biomedical and Neuromotor Science-DIBINEM, University of Bologna, Bologna, Italy; 3 Medical Technology Laboratory, IRCCS Istituto Ortopedico Rizzoli, Via Di Barbiano 1/10, 40136 Bologna, Italy

**Keywords:** Registry, THA, RIPO, Anatomic stem, Modular neck, Standard stem, Short neck

## Abstract

**Background:**

Anatomic stems for total hip arthroplasty (THA) have been developed to achieve a precise geometric fit between the implant and the surrounding femoral bone, aiming at the improvement of primary stability of cementless implants until osteointegration occurs. The aim of the current study is to go over the regional Registry of Orthopaedic Prosthetic Implants (RIPO) to analyze survivorship of THA implants when anatomic stems are used; moreover, separate analysis for modular and nonmodular stems, and in standard and short implants, is presented.

**Materials and methods:**

This retrospective registry study involved the analysis of data collected by the RIPO registry between 2000 and 2019. The study focused on THAs performed for primary hip osteoarthritis (OA) between 2000 and 2019. All patients treated by THA within this time frame and officially registered in the RIPO registry were included in the study. Exclusion criteria were: revision THAs, cemented implants, hemiarthroplasties, resurfacing procedures, megaprostheses for neoplastic and non-neoplastic conditions, and THAs performed on patients residing outside the region.

**Results:**

A total of 12,010 cementless primary THAs using curved anatomic stems were performed in Emilia-Romagna between 2000 and 2019 and formally registered in the RIPO registry. The overall survival rate for anatomic standard stems was 96.7% at 10 years (96.1–97.3%); at 15 from the surgery, the survival rate dropped to 95.1% (93.9–96.1%). A total of 473 out of 12,010 recorded THA with anatomic stems (3.93%) experienced failure requiring revision surgery. The fixed standard stem showed the lowest failure rate (0.6%), while modular short stems had the highest (7.4%) at long-term follow-up. The most common stem-related complication was periprosthetic fracture (PF) in short stems (2.0% of cases) while in standard stems it was implant breakage (0.9% of cases); PFs were significantly more frequent in female patients (*p* = 0.0082), with a relative risk (RR) of 1.59 compared with male patients. Implant breakage demonstrated the highest rate of incidence among standard-modular stems (1.1% of cases).

**Conclusions:**

This registry-based study highlights that stem length and modularity significantly affect the long-term survival of anatomic femoral stems in THA. Fixed standard stems had the lowest failure rates, while modular short stems showed the highest failure rates and complications.

*Level of evidence*: 3.

## Introduction

Since the introduction of modern total hip arthroplasty (THA) by Charnley in 1959 [1], advancements in femoral stem design have focused on improving length, shape, and fixation methods [2–4]. Cementless fixation dominates in primary THA, accounting for 86% of procedures in the USA and 87.4% in Italy, with even higher rates in younger patients [5–9]. Achieving precise geometric fit between the femoral component and bone is crucial for primary stability and successful osteointegration [2, 10, 11]. Anatomic stems were designed to mimic the proximal femur’s natural geometry, enhancing stability and reducing stress shielding and subsidence, with long-term survival rates exceeding 90% [2, 6, 10, 12–14].

In recent years, bone-preserving short stems, often with an anatomic design, have gained popularity. These stems aim to minimize stress shielding, reduce thigh pain, and simplify revision surgery by focusing fixation at the metaphysis [15–18]. Concurrently, modular stems have provided surgeons with greater intraoperative flexibility in adjusting limb length, offset, and anteversion [19–21]. However, complications such as corrosion and taper fractures have raised concerns [21, 22].

Despite these innovations, limited comparative data exist on long-term outcomes of anatomic stems on the basis of length and modularity. No large registry studies have addressed this, leaving a critical gap in understanding how these variables influence survival rates and complications. Addressing this gap, our registry study leverages data from the Emilia-Romagna Region Registry of Orthopaedic Prosthetic Implants (RIPO) to evaluate the impact of stem length and modularity on implant survival and failure causes. Identifying optimal stem designs could reduce implant failures and the socioeconomic burden of revision surgeries [23–26].

## Material and methods

This observational retrospective registry study involved the analysis of data collected by the Emilia-Romagna (ER, Italy) Registry of the Orthopaedic Prosthetic Implants (RIPO). Established in 1990, RIPO records nearly 98% of arthroplasty implants performed in the Emilia-Romagna Region, including procedures conducted in both national healthcare system and private orthopedic facilities, for a total of 62 participating hospitals.

The study focused on THAs performed for primary degenerative hip osteoarthritis (OA) between 2000 and 2019. All patients treated by THA within this time frame and officially registered in the RIPO registry were included in the study. There were no restrictions on the inclusion criteria for patients based on age and gender. The study focused exclusively on patients residing within the ER region to mitigate potential bias originated from loss at follow-up. As a result, any THA performed on patients residing outside ER were deliberately excluded from the analysis. Revision THAs, cemented implants, hemiarthroplasties, resurfacing procedures, and the use of megaprostheses for neoplastic and non-neoplastic conditions were also excluded.

Data extraction from the RIPO database was performed on 9 August 2023, and implant survival and failure were collected until 31 December 2019. RIPO standard reporting included stem manufacturer, implant model, and fixation, but it did not specify the geometric shape and the length of stems. Therefore, two researchers (M.B., V.R.) independently selected the curved anatomic stems among the stems recorded from the RIPO, further dividing these into standard and short according to the traditional 120 mm length cutoff [19]; moreover, for each stem the presence or absence of a neck modularity was reported. In case of disagreement, the senior author (A.D.M.) determined the most appropriate stem attribution.

The study considered several data, including patients’ age at surgery, sex, body mass index (BMI), and number of cementless anatomic femoral stems implanted in primary THAs during the study period, categorized according to their length and modularity. Implant survival was analyzed for each anatomic stem type, with failure defined as any surgery requiring revision of at least the femoral stem. All complications leading to the failure of femoral stems were analyzed. This comprehensive evaluation allowed us to assess, for each anatomic stem type, the percentage incidence and the magnitude of specific stem complications relative to the total causes of stem failure. Survival and complications were further analyzed on the basis of the presence of neck modularity, providing different stem version options.

Ethical approval was not required for this study, since data collection is an ER standard practice, and the identity of the patients is concealed. Furthermore, no adjunctive clinical procedures were performed besides the analysis of registry data.

### Statistical analysis

Descriptive statistics, such as median and range for continuous variables and frequency with percentage (%) for categorical variables were used for data report. The chi-squared test was employed to assess statistical significance of qualitative data, while the analysis of variance (ANOVA) test was used for continuous data. Kaplan–Meier survivorship analysis was performed using the revision of at least the femoral stem component as endpoint, with implant survival of non-revised THAs considered as the last date of observation (31 December 2019 or the date of death available from the ER database). The log-rank test was used to compare survivorship between groups. The Wald test was conducted to analyze the *p*-values for data achieved from the Cox multiple regression analyses. The proportional hazards assumption was estimated using the Schoenfeld residual method and *p*-values < 0.05 were considered significant. Statistical analyses were conducted using SPSS 14.0, version 14.0.1 (SPSS Inc., Chicago, IL, USA), and JMP, version 12.0.1 (SAS Institute Inc., Cary, NC, USA, 1989–2007).

## Results

### Participants

A total of 12,010 cementless primary THAs using curved anatomic stems were performed in ER between 2000 and 2019 and formally registered in the RIPO registry. All demographic characteristics of study participants are presented in Table 1

**Table 1:** Demographic characteristics of study participants, including age, gender, and BMI

	Anatomic stems divided by length		*p*-Value^b^
	Short, *N* = 6353^a^	Standard, *N* = 5657^a^	
Age			< 0.001
Median (range)	69.0 (24.0, 92.0)	70.0 (20.0, 96.0)	
Mean (SD)	67.9 (9.0)	68.7 (9.6)	
Age class			< 0.001
≤ 65	2322 (36.5)	1898 (33.6)	
> 65	4031 (63.5)	3759 (66.4)	
Gender			0.589
F	3665 (57.7)	3292 (58.2)	
M	2688 (42.3)	2365 (41.8)	
BMI			< 0.001
Underweight	31 (0.5)	31 (0.6)	
Normal	1494 (26.0)	1565 (29.4)	
Overweight	2886 (50.3)	2447 (46.0)	
Obese	1332 (23.2)	1274 (24.0)	
Unknown	610	340	

^a^*n* (%)

^b^Welch two sample *t*-test; Pearson’s chi-squared test

The most frequently implanted anatomic stem (Table 2) during the study period was the APTA Adler (Adler Ortho, Milan, Italy), standard stem with a modular neck, accounting for 5041 implants (89.1% of overall standard anatomic stems). The most frequently implanted short anatomic stem was the ABGII Howmedica (Stryker Orthopaedics, Portage, USA), presenting a fixed neck, and accounting for 2031/6353 implants (32.0% of overall short anatomic stems).

**Table 2:** Anatomic stems implanted during the study period

Anatomic stems divided by length	Fixed neck	Modular neck	Total (%)
Short	4122	2231	6353 (100.0)
ABGII howmedica	2031		2031 (32.0)
Anca fiT cremascoli		1980	1980 (31.2)
Minimax medacta	831		831 (13.1)
CFP link	707		707 (11.1)
ABG howmedica	192		192 (3.0)
Anato stryker orthopaedics	187		187 (2.9)
SPS symbios	174		174 (2.7)
SPS modular symbios		153	153 (2.4)
MBA hap groupe lepine		98	98 (1.5)
Standard	616	5041	5657 (100.0)
Apta adler non cem		5041	5041 (89.1)
Apta-fix adler	616		616 (10.9)
Total	4738	7272	12,010

Among the short anatomic stems, the use of a fixed neck was reported in 4122/6353 surgeries (64.9%), while a modular neck was used in 2231 cases (35.1%) (Table 3). Conversely, among standard anatomic stems, modular neck represented most of implants with 5041/5657 (89.1%), with fixed neck used in 616/5657 cases (10.9%).

**Table 3:** Neck modularity used for anatomic stems implanted during the study period

	Anatomic stems divided by length		*p*-Value^b^
	Short, *N* = 6353^a^	Standard, *N* = 5657^a^	
Neck			< 0.001
Fixed	4122 (64.9)	616 (10.9)	
Modular	2231 (35.1)	5041 (89.1)	

^a^*n* (%)

^b^Welch two sample *t*-test; Pearson’s chi-squared test

### Stem failures requiring revision

During the study period, a total of 473 out of 12,010 recorded THAs (3.93%) experienced failure requiring revision surgery. Table 4 provides a breakdown of the number of stem failures requiring revision surgery during the follow-up period, according to their length and modularity. During the follow-up period, short anatomic stems showed a higher incidence of stem failure (5.1%) compared with standard anatomic stems (2.6%); short-modular stems exhibited the highest incidence of stem failure, followed in decreasing order by short-fixed, standard-modular, and standard-fixed stems, which showed the lowest incidence of stem failure (0.6%).

**Table 4:** Quantitative analysis of stem failures requiring removal–revision surgery during the follow-up period, categorized according to length and neck modularity

Group	Number of stem failures	Non-failed stems	Incidence of stem failure (%)	Mean follow-up (years) (min–max)
Short	324	6029	5.1	11.4 (0–21)
Short-fixed	160	3962	3.9	10.1 (0–21)
Short-modular	164	2067	7.4	13.6 (0–21)
Standard	149	5508	2.6	7.6 (0–16)
Standard-fixed	4	612	0.6	2.9 (0–6)
Standard-modular	145	4896	2.9	8.2 (0–16)

### Incidence of intraoperative periprosthetic fractures

The incidence of intraoperative stem fractures was then analyzed by dividing them according to length and then according to fracture area (calcar, acetabulum, diaphysis). The most significant result concerns the incidence of intraoperative calcar fracture using the short stem, at 0.6% (Table 5).

**Table 5:** Quantitative analysis of intraoperative fractures during the follow-up period, categorized by stem length

Stem	Yes	No	Incidence of intraoperative fracture (%)
Short			
Calcar fracture	39	6314	0.6
Acetabular fracture	4	6349	0.06
Diaphyseal fracture	9	6344	0.14
Standard			
Calcar fracture	13	5644	0.2
Acetabular fracture	3	5654	0.05
Diaphyseal fracture	9	5648	0.16

### Survivorship analysis between stem types

#### Implant survival according to anatomic stems length

**Fig. 1:**
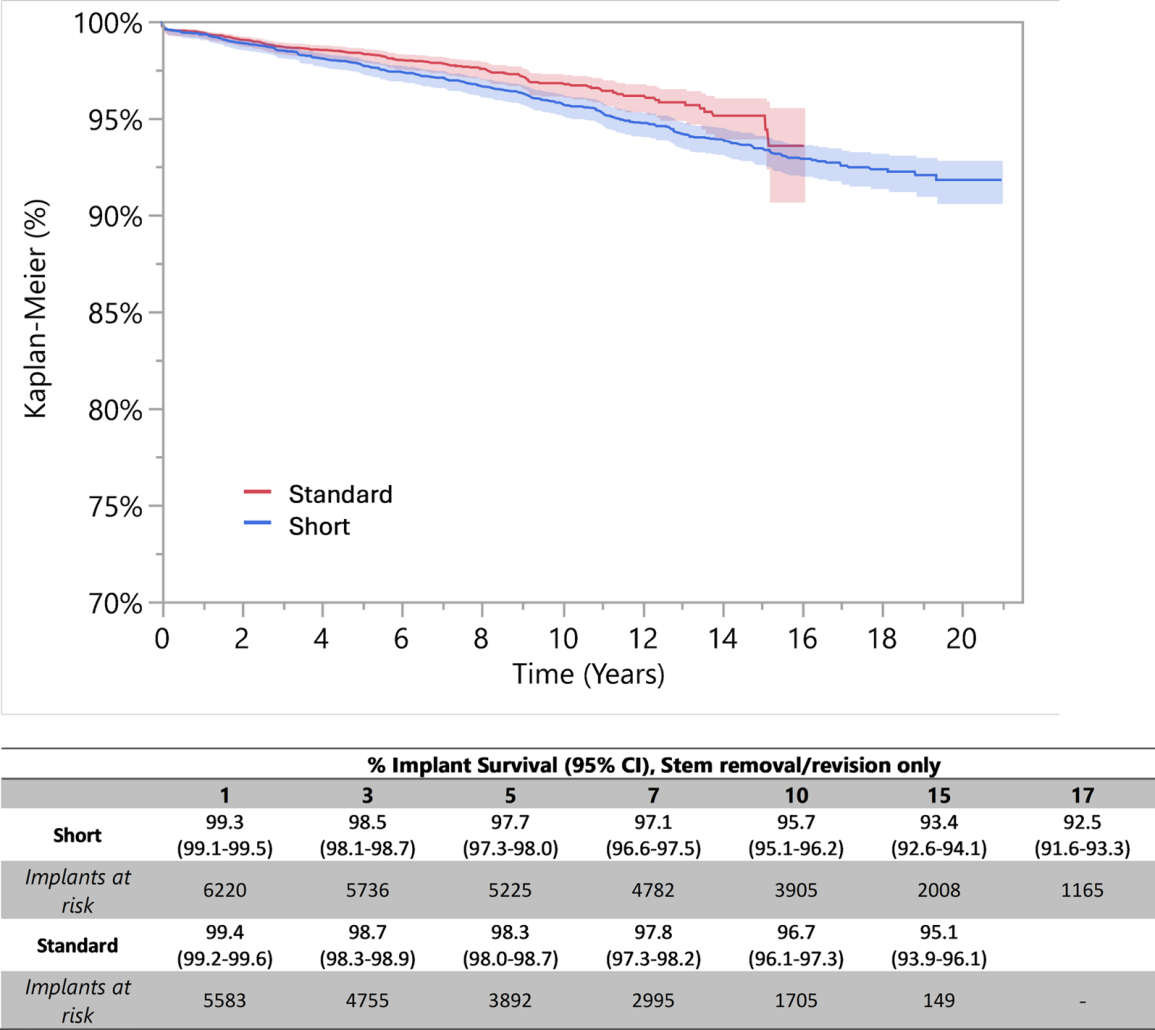
Implant survival according to stem implant length at 1, 3, 5, 7, 10, 15, and 17 years; % implant survival (95% CI), stem removal/revision only. Kaplan–Meier survivorship analysis between groups for stem removal-revision during the various follow-up periods, represented both as percentage of implant survival with ranges and graphically over time. In particular, the survival rate for anatomic short stems (blue line) was 95.7% at 10 years (95.1–96.2%); at 15 years, the survival rate averaged 93.4% (92.6–94.1%), decreasing to 92.5% at 17 years (91.6–93.3%). For anatomic long stems, the overall survival rate was 96.7% at 10 years (96.1–97.3%). At 15 years, the survival rate averaged 95.1% (93.9–96.1%). At 17 years, the survival rate for this group is missing due to implants at risk lost during the follow-up period

Kaplan–Meier survivorship analysis revealed different survival rates at follow-up of 1, 3, 5, 7, 10, 15, and 17 years among the recorded anatomic stems divided according to their length (Fig. 1).

Pairwise comparisons considering the entire follow-up period showed significant differences in survival curves between anatomic standard stems compared with anatomic short stems (*p* = 0.009), with standard stems showing higher survival compared with short stems throughout the entire follow-up period. Compared with anatomic standard stems, the relative risk (RR) of failure was 1.30 higher in patients with anatomic short stems, after adjusting for age and sex.

#### Implant survival according to anatomic stems length and modularity

**Fig. 2:**
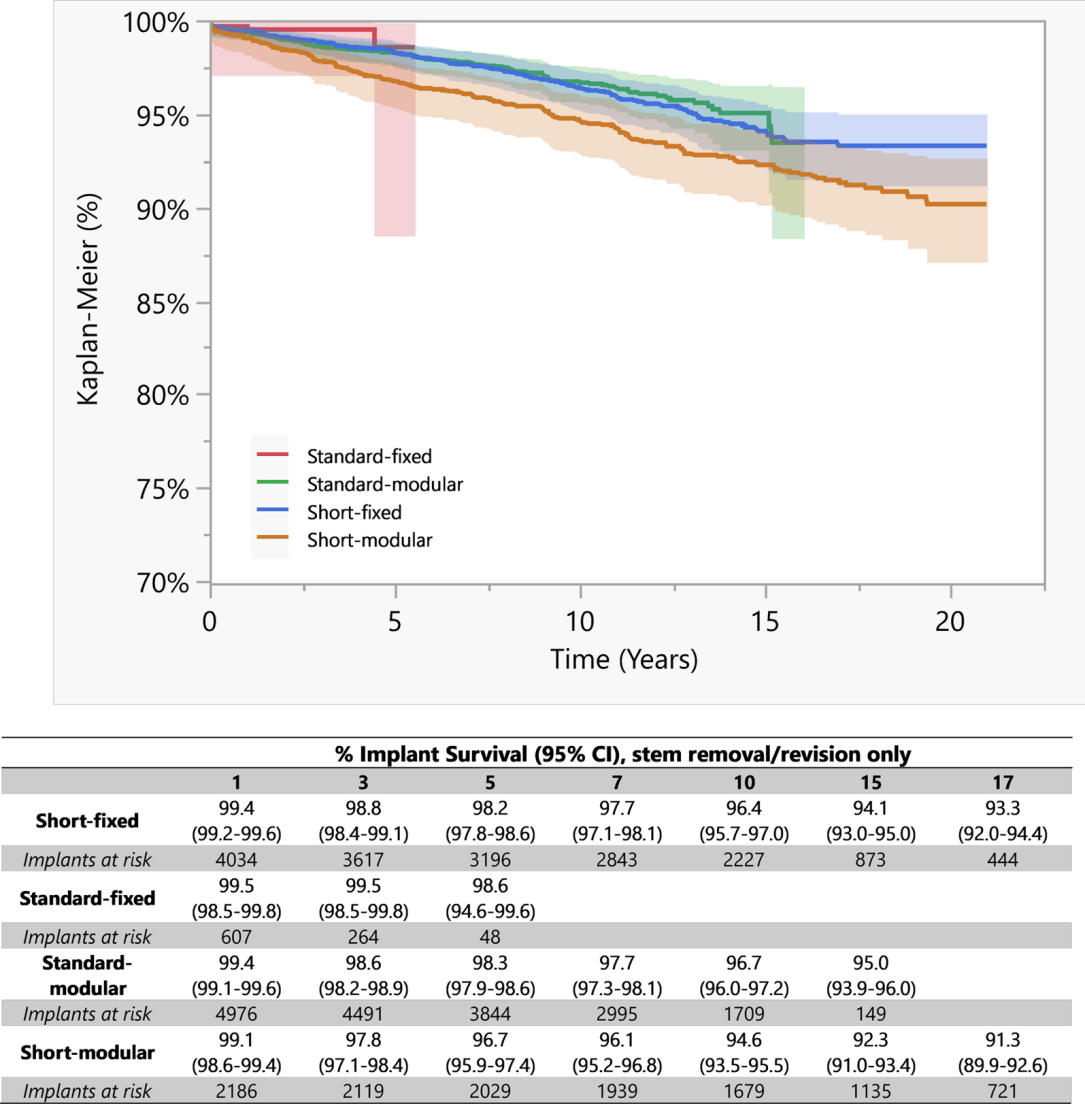
Implant survival at 1, 3, 5, 7, 10, 15, and 17 years. Kaplan–Meier survivorship analysis between groups for stem removal–revision during the various follow-up periods, represented both as percentage of implant survival with ranges and graphically over time. In particular, the survival rate for anatomic short-fixed stems was 96.4% at 10 years (95.7–97.0%); after 15 and 17 years from implant, the survival rate decreased to 94.1% (93.0–95.0%) and 93.3% (92.0–94.4%), respectively. For anatomic short-modular stems, the overall survival rate was 94.6% at 10 years (93.5–95.5%). At 15 years, the survival rate averaged 92.3% (91.0–93.4%). After 17 years, the survival rate was 91.3% (89.9–92.6%). For anatomic long-fixed stems, the overall survival rate was 98.6% at 5 years (94.6–99.6%). Long-term survival data at 10, 15, and 17 years are missing due to implants at risk lost during the follow-up period. Anatomic long-modular stems showed a survival rate of 96.7% at 10 years (96.0–97.2%) and 95.0% at 15 years (93.9–96.0%). Survival data at 17 years are missing due to implants at risk lost during the follow-up period

Kaplan–Meier survivorship analysis revealed different survival rates at follow-up of 1, 3, 5, 7, 10, 15, and 17 years among the recorded anatomic stems divided according to their length and modularity (Fig. 2).

Pairwise comparisons considering the entire follow-up period showed statistically significant differences in survival curves between standard-fixed stems compared with short-modular stems (*p* = 0.0271; RR 3.09 higher for short-modular stems), between standard-modular stems compared with short-modular stems (*p* = 0.0003; RR 1.53 higher for short-modular stems), and between short-fixed stems compared with short-modular stems (*p* = 0.0027; RR 1.41 higher for short-modular stems).

### Causes of stem failures

Periprosthetic fracture (PF) represented the most common cause of anatomic stem failure necessitating revision surgery in short stems, with an incidence of 2.0% and accounting for 38.6% of all failures. Conversely, implant breakage emerged as the primary cause of failure in standard stems, observed in 0.9% of cases (Table 6). Considering all anatomic stems in terms of length and modularity, the type of stem that recorded the highest incidence of PF was the short-fixed (2.0%), followed in descending order by short-modular, standard-modular, and lastly, the standard-fixed (0.5%).

**Table 6:** Causes and incidence of THA failure requiring revision divided in groups according to stem length and modularity

Cause of stem failure	Short				Standard			
		*N*	Incidence (%)	% cause of failure		*N*	Incidence (%)	% cause of failure
Pain without loosening	Total	7	0.1	2.2	Total	1	0.0	0.7
	Fixed	1	0.0	0.6	Fixed	–	0.0	0.0
	Modular	6	0.3	3.7	Modular	1	0.0	0.7
Periprosthetic fracture (PF)	Total	125	2.0	38.6	Total	38	0.7	25.5
	Fixed	82	2.0	51.3	Fixed	3	0.5	75.0
	Modular	43	1.9	26.2	Modular	35	0.7	24.1
Primary instability	Total	4	0.1	1.2	Total	2	0.0	1.3
	Fixed	3	0.1	1.9	Fixed	–	0.0	0.0
	Modular	1	0.0	0.6	Modular	2	0.0	1.4
Dislocation	Total	25	0.4	7.7	Total	14	0.2	9.4
	Fixed	6	0.1	3.8	Fixed	–	0.0	0.0
	Modular	19	0.9	11.6	Modular	14	0.3	9.7
Missing	Total	45	0.7	13.9	Total	10	0.2	6.7
	Fixed	28	0.7	17.5	Fixed	1	0.2	25.0
	Modular	17	0.8	10.4	Modular	9	0.2	6.2
Aseptic loosening (stem)	Total	63	1.0	19.4	Total	9	0.2	6.0
	Fixed	27	0.7	16.9	Fixed	–	0.0	0.0
	Modular	36	1.6	22.0	Modular	9	0.2	6.2
Septic loosening	Total	11	0.2	3.4	Total	11	0.2	7.4
	Fixed	4	0.1	2.5	Fixed	–	0.0	0.0
	Modular	7	0.3	4.3	Modular	11	0.2	7.6
Ossification	Total	1	0.0	0.3	Total	3	0.1	2.0
	Fixed	–	0.0	0.0	Fixed	–	0.0	0.0
	Modular	1	0.0	0.6	Modular	3	0.1	2.1
Implant breakage	Total	15	0.2	4.6	Total	53	0.9	35.6
	Fixed	2	0.0	1.3	Fixed	–	0.0	0.0
	Modular	13	0.6	7.9	Modular	53	1.1	36.6
Other	Total	6	0.1	1.9	Total	3	0.1	2.0
	Fixed	–	0.0	0.0	Fixed	–	0.0	0.0
	Modular	6	0.3	3.7	Modular	3	0.1	2.1

Pairwise comparisons considering the entire follow-up period showed significant differences in the incidence of PF between short-fixed stems compared with standard-modular stems (*p* = 0.0005; RR 2.04 higher for short-fixed stems), and between short-modular stems compared with standard-modular stems (*p* = 0.0243; RR 1.71 higher for short-modular stems).

Multivariate analysis showed a significant difference in the incidence of PF on the basis of patient gender (*p* = 0.0082), with female patients being more at risk of developing this complication with a RR of 1.59 compared with male patients.

Among the other causes of stem failure, aseptic loosening of the stem showed the highest incidence in short-modular stems (1.6% of cases, accounting for 22.0% of failures), as well as dislocation (0.9), septic loosening, and global aseptic loosening. Primary instability showed the highest incidence in short-fixed stems, whereas pain without loosening appeared more frequent in short-modular stems.

Implant breakage demonstrated the highest rate of incidence among standard-modular stems (1.1% of cases, accounting for 36.6% of failures), followed in descending order by short-modular, short-fixed, and lastly, standard-fixed, which showed no implant ruptures during the entire follow-up period. The modular neck of anatomic stems represented the most involved area of implant breakage, accounting for 72.1% of cases [46 recorded cases for standard-modular stems, entirely APTA-Adler (Adler Ortho, Milan, Italy); 3 recorded cases for short-modular stems, 1 Cremascoli Anca-fit (Wright Orthopedics Corp, MS, USA), and 2 SPS Modular Symbios (Symbios Orthopédie, Yverdon-les-Bains, Swiss)], representing the whole figures of ruptures of the stem component. Other causes of implant breakage included, in descending order, cup-inlay, femoral head, and combination of both.

Focusing on ruptures of the modular neck, which represent the most involved area of implant breakage, Cox multivariate analysis showed an increased risk of neck fracture in obese patients compared wit underweight–normal weight patients with RR of 7.47 (*p* = 0.0002) and compared with overweight patients with RR of 5.39 (*p* = 0.0001). Male patients showed a higher risk of implant breakage compared with female patients, with RR of 5.87 (*p* = 0.0001).

## Discussion

Our study revealed that the long-term survival rate differed significantly among various types of anatomic stems when categorized by length, with standard stems demonstrating significantly higher survival curves compared with short stems throughout the entire follow-up period (*p* = 0.009; RR 1.3 for short stems). Our data align with current literature, including large analyses such as the 2018 report from the Australian Orthopedic Association National Joint Replacement Registry [27], which described that the cumulative incidence of loosening for short-stemmed THAs was about twice that of standard-length femoral components at 10 years. However, that study, as well as most of the current literature, focused on survival and complication comparisons of standard versus short femoral stems only, without considering the specific stem design and shape. In the current study we only considered anatomic stems, for which there is still a lack of large comparative studies in literature.

Looking at individual studies, the results are conflicting compared with our report, with survival rates of short anatomic stems being extremely variable. Several authors reported excellent results; Nourissat et al. [28] described 90 consecutive primary anatomic short ABG II THAs, reporting a cumulative stem survival rate at 10 years of 98.7% ± 1.3%. Similarly, Kim et al. [17] in 2014 reported on 500 patients (630 THAs) operated on by a short metaphyseal-filling anatomic stem, and they found a 15-year survival rate of 99.4% (CI 98–100) for the femoral component. Furthermore, in 2021 they conducted a comparison study of 858 ultra-short anatomic stems and 858 standard-length anatomic stems [15], and found that the survival rate at 17 years of the ultra-short cementless anatomic stem (97.6% CI 94–100) was comparable to the standard-length cementless anatomic stem (96.6% CI 92–100). The same authors reported no significant differences in the survival of short, metaphyseal-fitting anatomic stems in different bone quality Dunn classes [29] at 7 years. Short and ultra-short anatomic femoral stems were also studied recently in patients younger than 30 years [18] affected by hip osteoarthritis secondary to avascular necrosis, and they reported a 17-year survival rates of the femoral component of 99.2% (CI 94–100).

In the current study, stem modularity as well as length were compared; we found significant differences in terms of survival comparing standard-fixed stems to short-modular stems (*p* = 0.0271; RR 3.09 for short-modular stems), standard-modular stems to short-modular stems (*p* = 0.0003; RR 1.53 for short-modular stems), and short-fixed stems to short-modular stems (*p* = 0.0027; RR 1.41 for short-modular stems). Our data align with the recent literature confirming that the presence of neck modularity in primary THAs negatively affects the long-term survival of the femoral component. For instance, Colas et al. [30] in 2017 conducted a study on a French Nationwide Cohort of 324,108 patients, identifying a total of 8931 (3%) patients with exchangeable neck stem implants. They found that these modular implants were more likely to undergo revision compared with fixed neck stem designs (RR 1.36; CI, 1.24–1.49; *p* < 0.001).

Most of current literature focus on survival and complication comparisons of modular versus fixed femoral stems, without considering the specific stem design and shape. When the anatomic design is specifically considered, there is a void in current literature. Looking at individual studies, survival rates of modular anatomic stems are extremely variable, but still show survival rates exceeding 90%, especially for standard stems. Castagnini et al. [11] described a registry cohort of 1984 standard-modular anatomic stems with a reported 9-year survival of 98.6% (CI 97.9–99%). Similarly, in a 2017 registry study, Toni et al. [31] reported on 300 THAs with standard-modular anatomic stems, finding a 15-year survival rate of 97.2% (95% CI 94.8–100%). Regarding short-modular anatomic stems, Mouttet et al. [32] reported a series of 176 THAs using these stems, showing an excellent 5-year survival of the femoral component of 98.8%; however, at last follow-up, survival decreased to 93.2%. Tostain et al. [33] included 61 primary THAs with anatomical short-modular stems, reporting a 10-year survival rate of 96% (CI 88–99%). Cossetto et al. [34] reported on 185 THAs with short-modular anatomic stems, showing a 10-year survival of 99% (CI 97–100%).

In our cohort, the most frequent stem-related complication was periprosthetic fracture (PF) in short stems (2.0% of cases, accounting for 38.6% of failures). The type of stem that recorded the highest incidence of PF was the short-fixed (2.0% of cases, accounting for 51.3% of failures); these were significantly more frequent in female patients (*p* = 0.0082), with a relative risk (RR) of 1.59 compared with males. Pairwise comparisons showed statistically significant differences in the incidence of PF between short-fixed stems compared with standard-modular stems (*p* = 0.0005; RR 2.04) and between short-modular stems compared with standard-modular stems (*p* = 0.0243; RR 1.71). In a 2024 study performed by Turnbull’s group regarding the survival of 1000 consecutive Lubinus SP2 anatomic stem implants (standard anatomic stem with fixed neck) the incidence of periprosthetic fractures was 0.3%, which is consistent with the 0.5% reported in the current study [12]. In a study involving 496 short-modular anatomic stems (ESOP stem, FH^®^), Martínez Martín et al. [35] showed a periprosthetic fracture rate of 3.3%, a rate almost double compared with our findings (1.9%), supporting the decreased use of anatomic stems with full modularity (i.e., diaphyseal and metaphyseal).

Regarding data on modular neck fractures in anatomical stems, literature is mostly focused on neck fractures in non-anatomic implants; the trend of use of stems with neck modularity appears to be decreasing overtime, as outlined by the latest Australian registry report in 2023 [36]. However, modular necks are frequently used by orthopedic surgeons worldwide, and the knowledge that obese male patients are less suitable for the use of these implants may be of support for implant choice.

There are several limitations to our research. First, it is retrospective and relies on observational data. As a result, it was not possible to establish cause–effect relationships or to evaluate individual factors that may have confounding effects. Moreover, intrinsic to the nature of the registry, it was not possible to estimate preoperative conditions, such as disease severity, functional aspects, and postoperative outcomes. Survivorship analyses are incomplete due to the loss of implants at risk during follow-up period, and the analysis based on periprosthetic fractures were limited to two stem types (standard-fixed and short-modular) due to an insufficient number of cases. Further, this is the first registry study that examines the survivorship of anatomical stems on the basis of their length and modularity, including an analysis of the specific causes of failure. Future prospective research should look at the overall survival of prosthetic stems while considering patient lifestyle and physical activity rate. The initial choice of stem implants is critical to the long-term success of THA surgery, and the findings of this study are useful for optimizing implant selection; the most important clinical application arising from this study is the support of orthopedic surgeons during the selection of femoral stem implants in the preoperative planning of THA procedures.

## Conclusions

This registry-based study provides robust evidence regarding the long-term survival of anatomic femoral stems in primary total hip arthroplasty. Our findings indicate that stem length and modularity are significant factors influencing implant survival. Specifically, anatomic stems demonstrated overall optimal survival rates. The fixed standard stem showed the lowest failure rate, while modular short stems had the highest at long-term follow-up. Modular neck designs, while offering flexibility in surgical adjustment, were associated with a higher incidence of complications, including implant breakage at the modular interface. These results suggest that standard length anatomic stems may be considered a useful stem in THA, though caution is advised when using modular stems, in particular short-anatomic, due to their associated risks.

## Data Availability

Not applicable.
